# The landscape of RNA polymerase II transcription initiation in *C. elegans* reveals a novel enhancer architecture

**DOI:** 10.1186/1756-8935-6-S1-O21

**Published:** 2013-03-18

**Authors:** Ron Chen, Thomas Down, Julie Ahringer

**Affiliations:** 1 The Gurdon Institute, University of Cambridge, Cambridge, UK

## 

RNA polymerase transcription initiation sites are largely unknown in *C. elegans.* The initial 5’ end of most protein-coding transcripts are removed by trans-splicing, and non-coding initiation sites have not been investigated. We characterized the global landscape of RNA Pol II transcription initiation, identifying 73,500 distinct clusters of initiation, many of which are bidirectional. We assign transcription initiation sites to 7691 protein-coding genes and find that initiation units show a range of patterns that have differences in core promoter elements and nucleosome positioning. We also show that HOT (high-occupancy target) regions bound by most transcription factors are core promoters for protein coding genes. Strikingly, the majority of transcription initiation events occur in regions with enhancer-like chromatin signatures. These define 3,405 putative enhancer regions (PERs), which are enriched at developmental and neuronal genes. The majority of PERs contain mapped transcription factor binding sites, and these often initiate bidirectional transcription. Remarkably, productive transcription elongation across PERs is usually directional, predominantly in the same orientation as that of the nearest downstream gene. We propose that this architecture is a feature of active enhancers.

